# The first record of the parasitic myrmecophilous caterpillar *Liphyrabrassolis* (Lepidoptera, Lycaenidae) inside Asian weaver ant (*Oecophyllasmaragdina*) nests in oil palm plantations

**DOI:** 10.3897/BDJ.10.e83842

**Published:** 2022-08-15

**Authors:** Andreas Dwi Advento, Kalsum M Yusah, Hasber Salim, Mohammad Naim, Jean-Pierre Caliman, Tom Maurice Fayle

**Affiliations:** 1 Smart Research Institute, Pekanbaru, Indonesia Smart Research Institute Pekanbaru Indonesia; 2 Institute for Tropical Biology and Conservation, Universiti Malaysia Sabah, Kota Kinabalu, Malaysia Institute for Tropical Biology and Conservation, Universiti Malaysia Sabah Kota Kinabalu Malaysia; 3 Royal Botanic Gardens Kew, London, United Kingdom Royal Botanic Gardens Kew London United Kingdom; 4 School of Biological Sciences, Universiti Sains Malaysia, 11800, Minden Penang, Penang, Malaysia School of Biological Sciences, Universiti Sains Malaysia, 11800, Minden Penang Penang Malaysia; 5 Biology Centre of the Czech Academy of Sciences, Institute of Entomology, Branišovská 1160/31, 370 05, České Budějovice, Czech Republic Biology Centre of the Czech Academy of Sciences, Institute of Entomology, Branišovská 1160/31, 370 05 České Budějovice Czech Republic; 6 School of Biological and Behavioural Sciences, Queen Mary University of London, London, United Kingdom School of Biological and Behavioural Sciences, Queen Mary University of London London United Kingdom

**Keywords:** Formicidae, parasitism, biocontrol, bagworm, Riau

## Abstract

Asian weaver ants (*Oecophyllasmaragdina*) are an important biocontrol agent in agricultural habitats. We conducted surveys in oil palm plantations in Riau, Indonesia for an obligate myrmecophilous butterfly larvae, *Liphyrabrassolis* (Lepidoptera, Lycaenidae), that is known to consume weaver ant larvae in other habitat types. We found *L.brassolis* larvae in five of the twenty nests surveyed, with larval presence not being related to weaver ant nest size. We also observed *L.brassolis* larvae in a weaver ant mass rearing facility. This is the first report of *L.brassolis* from oil palm plantations and may have implications for the use of weaver ants as biological control agents.

## Introduction

The Asian weaver ant, *Oecophyllasmaragdina* (Fabricius, 1775), is widespread from western Asia to northern Australia (Wetterer 2017) and maintains arboreal territories through aggressive behaviour. Worker ants patrol tree branches and attack other animals because they are generalist predators, preying on insects and other arthropods and also attack humans who disturb their nests (Crozier et al. 2010). The Asian weaver ant has several ecological and economic functions regarding its ecological interactions: enhancing potential nitrogen (Pinkalski et al. 2015, Vidkjær et al. 2015, Vidkjær et al. 2016), shaping plant-pollinator interactions (Rodríguez-Gironés et al. 2013) and acting as biocontrol agent (Peng et al. 1999, Peng and Christian 2005, Peng and Christian 2013, William et al. 2015, Anato et al. 2017). Weaver ants are commonly used as biocontrol agents in several agricultural systems with potential to control major pests such as true bugs (Coreidae and Miridae), beetles (Chrysomelidae), aphids (Aphididae), caterpillars (Lepidoptera), leaf miners (Coleoptera), leafrollers (Lepidoptera), fruit flies (Drosophilidae), leafhoppers (Cicadellidae) and shoot borers (Lepidoptera) (Van Mele 2007).

The majority of the species in the butterfly family Lycaenidae are associated with ants, either mutualistically or parasitically, with interactions either being facultative or obligate (Pierce et al. 2002). Parasitic interactions usually involve myrmecophagy, with butterfly larvae consuming the ant brood. In the family Lycaenidae, 74 species show this behaviour during some stage of their life cycle (Pierce 1995). These parasitic-myrmecophagy interactions can be found between Africa and northern Australia (Fiedler 1991, Eastwood and Fraser 1999, Pierce et al. 2002, Terblanche and Van Hamburg 2003, Crozier et al. 2010, Table 1).

*Liphyrabrassolis* (Westwood, 1864), is obligately myrmecophilous, with larvae being dependent on weaver ants (*Oecophyllasmaragdina*) and eating the ant brood (Dodd 1902, Eastwood and Fraser 1999, Pierce et al. 2002). As *L.brassolis* feeds on the larvae of weaver ants, it might have the potential to affect ant populations. Hence, it is important to understand its occurrence in widespread crops, such as oil palm plantation, where *O.smaragdina* has the potential to control pests, such as leaf-eating caterpillars.

## Materials and Methods

Sampling of weaver ant nests was conducted in an oil palm plantation located in the Province of Riau in Sumatra, Indonesia (0°56'35.3"N, 101°11'56.4"E) in August 2021. A localised outbreak of *Claniatertia* Templeton, 1847 (Lepidoptera, Pyschidae) bagworm was ongoing when the sampling was conducted. Twenty active weaver ant nests were chosen randomly on twenty individual palms. Only nests in which ants were present (confirmed using binoculars), constructed from live green palm leaflets, were sampled. The nest was collected, wrapped in a plastic bag and all connecting leaflets and fronds were cut using machete and pruning shears. Nest dimensions (length, width and depth) were measured. On the ground, nests were thoroughly dissected and checked for the presence of *L.brassolis* larva. Dissected nests were removed from bags and replaced at the base of the palm to allow ants to return.

During this period, as part of another study, we were conducting mass-rearing of weaver ants with the aim of propagation and release as biocontrol agent against leaf-eating caterpillar pests. All the colonies were fed on a 30% sugar solution and live insects or fish as a protein source. Observations of *L.brassolis* were conducted in this mass-rearing activity in 25 ant colonies that were being propagated in plastic bottles (Offenberg 2014) for integrated pest management (IPM).

*Liphyrabrassolis* larvae, found in ant nests, were identified morphologically (Crozier et al. 2010, Eastwood et al. 2010, Dupont et al. 2016). The larva of this species is distinguished by its carapace-like structure with a hard cuticle both dorsally and laterally (Fig. 1), which is used to defend against ants attacks (Dupont et al. 2016). All speciments of *L.brassolis* larvae and *O.smaragdina* were housed in Smart Research Institute, Riau, Indonesia.

A generalised linear model (GLM) with binomial errors was used to investigate the relationship between the weaver ant nest size and *L.brassolis* occurrence. The occurrence of *L.brasollis* larvae was used as a binary response variable with one nest sample per observation. Nest size was calculated as an ellipsoid volume using the three measured nest dimensions. Correction for overdispersion was conducted using the quasibinomial family since the residual deviance was larger than the degree of freedom (Crawley 2015). All statistical analyses were performed using the R statistical program version 4.0.2 (R Core Team 2020) in the R Studio version 1.1.423 (RStudio Team 2020) environment. Graphs were created using “*ggplot2*” and “*tidyverse*” R packages (Wickham et al. 2019). The generalised linear model was performed using the "*lme4*" package (Bates et al. 2015). The dataset of observation is deposited in GBIF, Global Biodiversity Information Facility (https://doi.org/10.15468/upzkak).

## Results and Discussion

**Figure 2.**
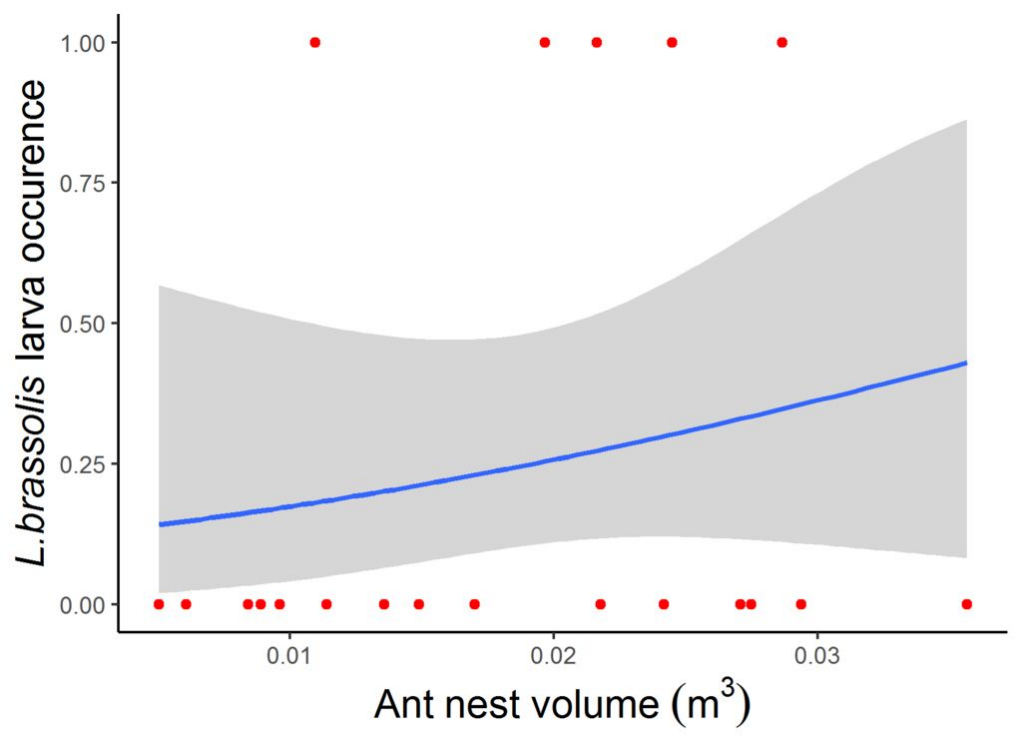
Relationship between *Liphyrabrassolis* larvae occurrence and weaver ant nest volume. Fitted line comes from a GLM with binomial errors.

*Liphyrabrassolis* larvae were present inside both weaver ant nests from the field (5 of 20 nests sampled) and mass-rearing observations (2 of 25 nests). In all seven nests with *L.brassolis* larvae present, only a single larvae was found (Suppl. material 1). A similar rate of occurrence of *L.brassolis* was reported during the ant harvesting season in Khao Chong, Thailand (this study: 16%, Eastwood et al. 2010: 21%). We found no evidence for a relationship between ant nest volume and *L.brassolis* larva presence (GLM, binomial errors, d.f. = 19, t = 0.78, p = 0.44; Fig. 2). This contrasts with a previous anecdotal observation mentioning that *L.brassolis* was found in larger weaver ant nests (Itterbeeck 2014).

**Figure 3.**
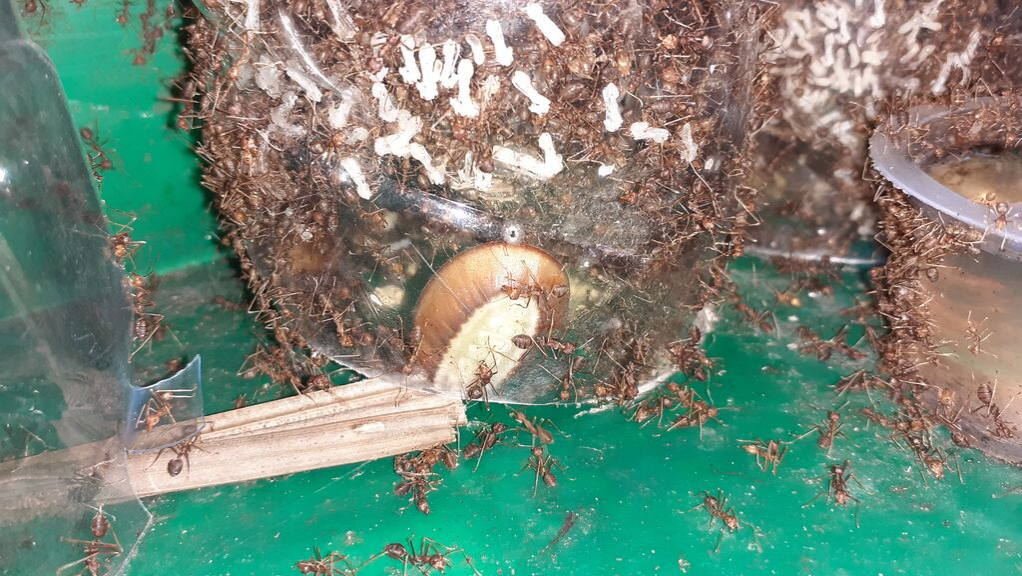
Presence of *Liphyrabrassolis* (Lepidoptera: Lycaenidae), an obligate-parasitic myrmecophily larva, inside a weaver ant (*Oecophyllasmaragdina*) colony in the mass-rearing facility.

We observed the larvae in the mass-rearing facility actively following the weaver ants during migration from the original disturbed leaf nest into the plastic bottle used to house the colony (Fig. 3). The presence of *L.brassolis* larvae in mass-rearing facilities should be avoided since consumption will reduce the number of ant brood. Mass-rearing operators should be trained to notice the *L.brassolis* larvae, so that larvae can be removed before the ant colony becomes established in the new nest. Education is needed here, since often local people misidentify *L.brassolis* larvae as a large ant or queen ant (Eastwood et al. 2010) . This is important because the weaver ant mass-rearing is commonly used to produce ant brood for use as bird feed (Prayoga 2015), a protein source for human consumption (Sribandit et al. 2008) or as an augmentation strategy for integrated pest management (IPM) programmes (Peng et al. 2014).

Although adult *L.brassolis* have been reported in West Java, Indonesia (Peggie 2012), this is the first report of this species in an oil palm plantation to our knowledge, despite multiple other butterfly surveys in this habitat in Sumatra (Panjaitan et al. 2020, Panjaitan et al. 2021). In Thailand, the larva of *L.brassolis* is commonly reported in weaver ant nests on orchard plantations, being first documented during entomophagy by local people (Eastwood et al. 2010). Furthermore, the presence of *L.brassolis* larva in weaver ant nests has not yet been reported during brood harvesting activity by local people in Indonesia (Césard 2004).

Implementation of biological control against leaf-eating caterpillars in an oil plantation is needed to support sustainable palm oil practices. However, only a small number of studies have investigated the ecological function of *O.smaragdina* as a biological control agent in oil palm plantation (Pierre and Idris 2013). With specific interaction with *O.smaragdina*, the butterfly *L.brassolis* can be a bioindicator for weaver ant colony in oil palm plantations. *Liphyrabrassolis* larvae are only ever found associated with *O.smaragdina*. However, other Lycaenid larvae have been observed in the nests of *Oecophylla* spp, such as *Liphyragrandis* and *Zesiuschrysomallus* (Pierce et al. 2002).

Our finding indicates that further investigations of weaver ant symbionts in oil palm plantations could be useful, in particular, because this ant species has potency for controlling leaf-eating caterpillar pests. It would be worth conducting larger-scale observations over greater numbers of colonies in order to measure any potential impacts of *L.brassolis* larvae on colony fitness, both in the field and in mass-rearing facilities. Furthermore, this observation represents an additional data point for lepidopteran biodiversity in Sumatra, especially in oil palm landscapes.

## Supplementary Material

64E71E8A-8F29-55D3-9D37-B133086F8588 10.3897/BDJ.10.e83842.suppl1 Supplementary material 1 *L.brassolis* field observation Data type Observation data Brief description These data are an observational census for *L.brassolis* occurrence in an oil palm plantation. They cover date sampling, location (plantation block), time sampling, nest dimension, larva number per nest and larva condition. (https://doi.org/10.15468/upzkak) File: oo_683034.xlsx https://binary.pensoft.net/file/683034 Andreas Dwi Advento

## Figures and Tables

**Figure 1a.**
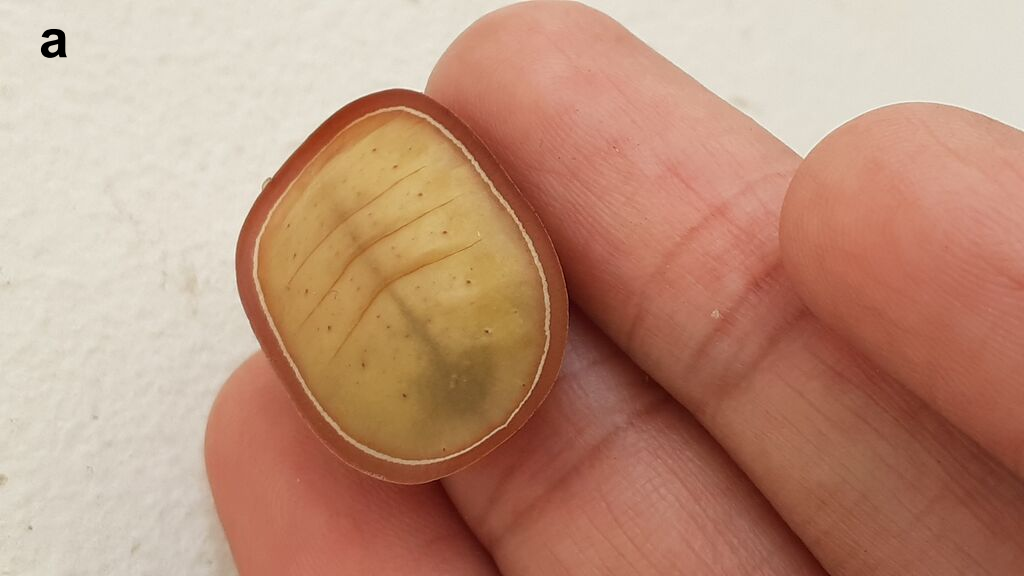
dorsal side of larva

**Figure 1b.**
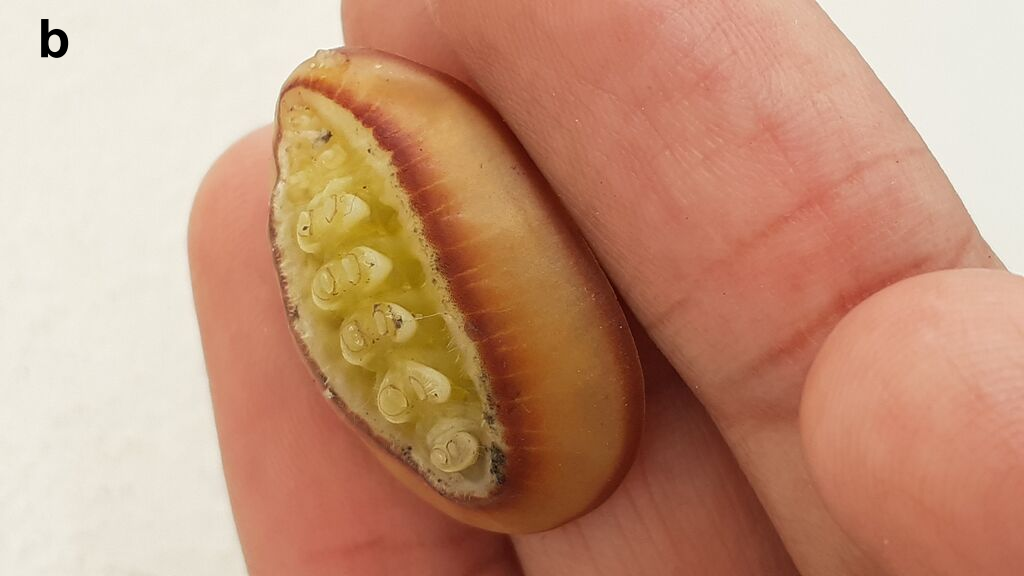
ventral side of larva

**Figure 1c.**
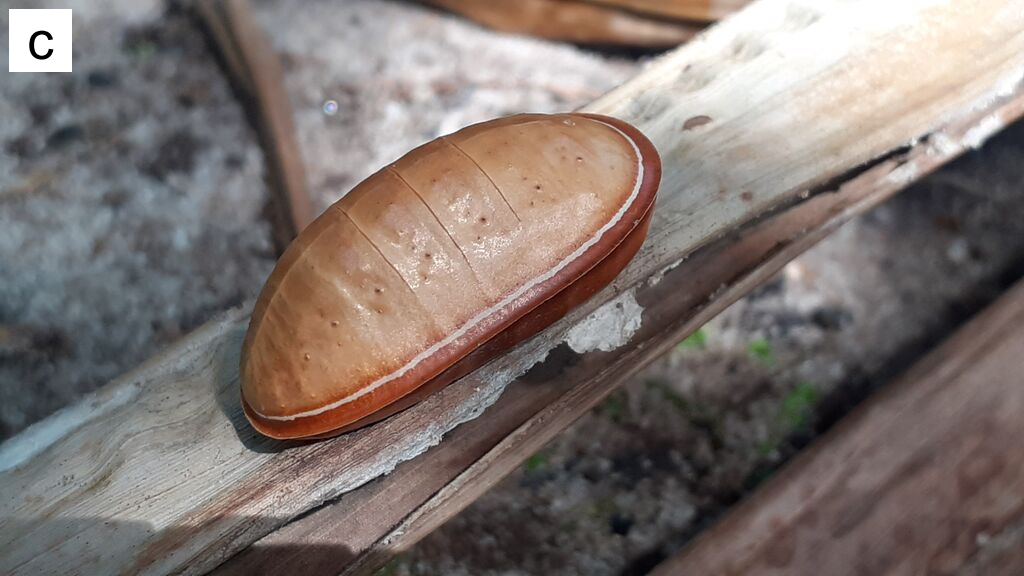
pupal stage

**Figure 1d.**
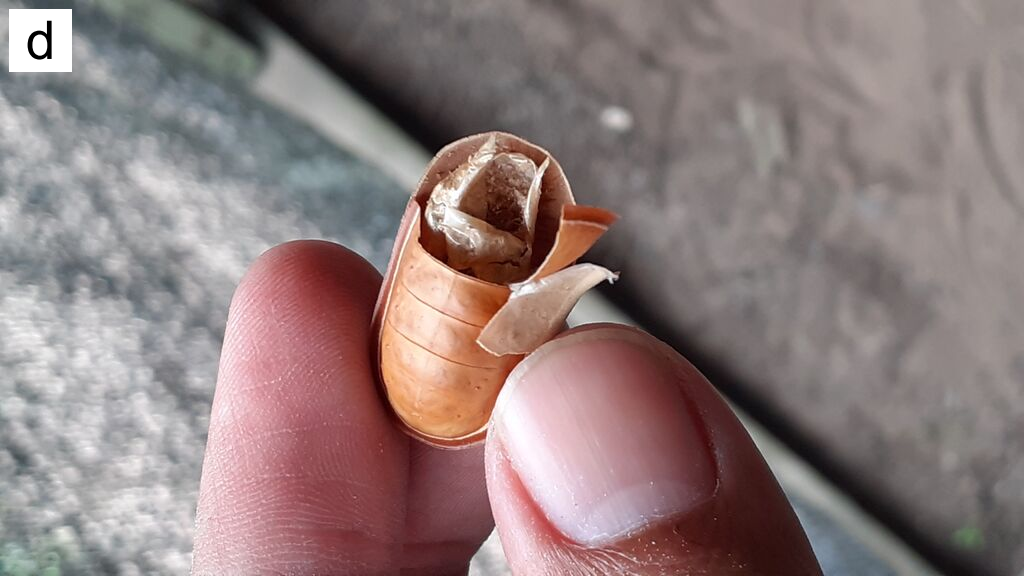
pupal exuvia

**Table 1.** List of lycaenid lepidopteran with myrmecophagy association

Family	Subfamily	Tribe/Subtribe	Species	Associated ant	Distribution	References
Lycaenidae	Miletinae	Liphyrini	*Liphyrabrassolis*	*Oecophyllasmaragdina*	Australia, Thailand, Malaysia, Indonesia	Fiedler (1991), Eastwood and Fraser (1999), Pierce et al.(2002), Crozier et al. (2010)
Lycaenidae	Miletinae	Liphyrini	*Liphyragrandis*	*Oecophylla* spp.	Papua, Australia, Pacific islands	Pierce et al.(2002), Crozier et al. (2010)
Lycaenidae	Miletinae	Lachnocnemini	*Lachonocnemabibulus*	*Camponotus* spp., *Crematogaster* spp., *Pheidole* spp.	Africa continent	Pierce et al. (2002)
Lycaenidae	Miletinae	Lachnocnemini	*Lachonocnemabrimo*	*Camponotus* spp., *Crematogaster* spp., *Pheidole* spp.	Africa continent	Pierce et al. (2002)
Lycaenidae	Miletinae	Lachnocnemini	*Lachonocnemadurbani*	*Camponotus* spp., *Crematogaster* spp., *Pheidole* spp.	Africa continent	Pierce et al. (2002)
Lycaenidae	Lycaeninae	Theclini/ Arhopalitii	*Arhopalawildei*	*Polyrachis* spp.	Papua, Northern Australia	Eastwood and Fraser (1999), Pierce et al. (2002)
Lycaenidae	Lycaeninae	Theclini/ Luciti	*Acrodipsasaurata*	*Crematogaster* spp., *Papyrius* spp.	Australia	Eastwood and Fraser (1999), Pierce et al. (2002)
Lycaenidae	Lycaeninae	Theclini/ Luciti	*Acrodipsasbrisbanensis*	*Crematogaster* spp., *Papyrius* spp.	Australia	Eastwood and Fraser (1999), Pierce et al. (2002)
Lycaenidae	Lycaeninae	Theclini/ Luciti	*Acrodipsascuprea*	*Crematogaster* spp., *Papyrius* spp.	Australia	Fiedler (1991), Eastwood and Fraser (1999), Pierce et al. (2002)
Lycaenidae	Lycaeninae	Theclini/ Luciti	*Acrodipsasillidgei*	*Crematogaster* spp., *Papyrius* spp.	Australia	Fiedler (1991), Eastwood and Fraser (1999), Pierce et al. (2002)
Lycaenidae	Lycaeninae	Theclini/ Luciti	*Acrodipsasmyrmecophila*	*Crematogaster* spp., *Papyrius* spp., *Iridomyrmex* spp.	Australia	Fiedler (1991), Eastwood and Fraser (1999), Pierce et al. (2002)
Lycaenidae	Lycaeninae	Theclini/ Zesiiti	*Zesiuschrysomallus*	*Oecophylla* spp.	South Asia	Pierce et al. (2002)
Lycaenidae	Lycaeninae	Theclini/ Ogyriti	*Ogyrisidmo*	*Camponotus* spp.	Australia	Fiedler (1991), Eastwood and Fraser (1999)
Lycaenidae	Lycaeninae	Aphnaeini	Cigaritis [Apharitis] acamas	*Crematogaster* spp.	Africa continent, Japan	Fiedler (1991), Pierce et al. (2002)
Lycaenidae	Lycaeninae	Aphnaeini	C. [Spindasis] japanesenyassae	*Crematogaster* spp.	Africa continent, Japan	Pierce et al. (2002)
Lycaenidae	Lycaeninae	Aphnaeini	C. [Spindasis] takanonis	*Crematogaster* spp.	Africa continent, Japan	Pierce et al. (2002)
Lycaenidae	Lycaeninae	Aphnaeini	*Trimeniaagyroplaga*	*Anoplolepis* spp.	Africa continent	Pierce et al. (2002)
Lycaenidae	Lycaeninae	Aphnaeini	*T.wallengrenii*	*Anoplolepis* spp.	Africa continent	Pierce et al. (2002)
Lycaenidae	Lycaeninae	Aphnaeini	T. [Argyrocupha] malagrida	*Anoplolepis* spp.	Africa continent	Pierce et al. (2002)
Lycaenidae	Lycaeninae	Aphnaeini	*Oxychaetadicksoni*	*Crematogaster* spp., *Myrmicaria* spp.	South Africa	Fiedler (1991), Terblanche and Van Hamburg (2003)
Lycaenidae	Lycaeninae	Polyommatini/ Polyommatiti	*Phengarisdaitozana*	*Myrmica* spp.	Asia	Pierce et al. (2002)
Lycaenidae	Lycaeninae	Polyommatini/ Polyommatiti	*P.atroguttata*	*Myrmica* spp.	Asia	Pierce et al. (2002)
Lycaenidae	Lycaeninae	Polyommatini/ Polyommatiti	*Lepidochrysops* spp.	*Camponotus* spp.	Africa continent	Pierce et al. (2002)
Lycaenidae	Lycaeninae	Polyommatini/ Polyommatiti	*Maculinea* spp.	*Myrmica* spp., *Aphaenogaster* spp.	Europe, Asia	Pierce et al. (2002)
